# Cancer-associated fibroblasts enhance tumor-associated macrophages enrichment and suppress NK cells function in colorectal cancer

**DOI:** 10.1038/s41419-019-1435-2

**Published:** 2019-03-20

**Authors:** Rongsheng Zhang, Fan Qi, Fei Zhao, Geng Li, Shengli Shao, Xiaochao Zhang, Lifei Yuan, Yongdong Feng

**Affiliations:** 1 Cancer Research Institute, Tongji Hospital, Tongji Medical College, Huazhong University of Science and Technology, Wuhan, Hubei 430030 China; 2 Department of Otolaryngology-Head and Neck Surgery, Tongji Hospital, Tongji Medical College, Huazhong University of Science and Technology, Wuhan, Hubei 430030 China; 3 Jinzhou Medical University, Liaoning, Jinzhou, 121000 China

## Abstract

Cancer-associated fibroblasts (CAFs) and tumor-associated macrophages (TAMs) are important components of the tumor microenvironment, which have been reported to localize in colorectal carcinomas where they promote tumor progression. One of the crucial effects they exerted is immune-suppression, which was reported recently, however, the overall mechanism has not been fully addressed. In this study, it was shown that TAMs were enriched in colorectal cancer, and their infiltration was associated with VCAM-1 expression. Human colorectal cancer-derived CAFs can promote the adhesion of monocytes by up-regulating VCAM-1 expression in colorectal cancer cells. Furthermore, CAFs can attract monocytes by secreting IL-8 rather than SDF-1 and subsequently promote M2 polarization of macrophages, which synergize with CAFs in suppressing the functioning of natural killer (NK) cells. It was also found that CAFs promoted M2 macrophages recruitment in tumor tissue in vivo, and after VCAM-1 knocking-down in tumor cells or depletion of macrophages, the pro-tumor effect of CAFs was partly abolished, but no change was observed in NK cells infiltration. Collectively, the findings in this work show that TAMs and CAFs function synergistically in the tumor microenvironment and have the capacity to regulate NK cells in colorectal cancer and this presents a novel mechanism.

## Introduction

Tumor cells are surrounded by stroma, which consists of various inflammatory cells, endothelial cell, and fibroblasts. Evolving crosstalk between different cells results in the progression of tumor, however the underlying mechanism is complex and remains obscure.

Cancer-associated fibroblasts (CAFs) are abundant stromal cells in the tumor microenvironment, they secrete cytokines such as HGF, IL-6, and SDF-1, which have a variety of effects on cancer cells and stroma^1–8^, including angiogenesis, tumor growth, migration, and are an alternative for extracellular matrix. Tumor-associated macrophages (TAMs), which are also dominant cell type in the tumor milieu, are estimated to account for up to 50% of cancer tissue mass^9^. There are two types of polarized macrophages: classically activated (M1) macrophages which activate immune response^10,11^ and alternatively activated (M2) macrophages which promote tumor progression^12–15^. The properties of TAMs are similar to those of M2 polarized macrophages.

Natural killer (NK) cells play an important role in the innate immune system, they can kill tumor cells without the necessity of prior sensitization^16^, NK-92 is a continuously expanding cytotoxic NK cells line which is under development for clinical application^17,18^. NK cells have advantages over T cell in their use for CAR-targeted immunotherapy, because they do not have MHC restriction, thus are not responsible for graft-versus-host disease (GVHD). Despite their therapeutic potential, NK cells therapies have limited success due to the suppression of tumor microenvironment. It has been reported that NK cells are no longer lyse tumor cells and express lower levels of activating receptor NKG2D after adoptive transfer^19^, which indicates that cells other than tumor cell suppress the function of NK cells in tumor microenvironment. Recently, it was reported that the immune-suppression role was played by CAFs or TAMs, but the overall mechanism remains obscure.

In the present study, colorectal cancer-derived CAFs (CC-CAFs) were isolated from human colorectal cancer (CRC) tissue, after which their effect on adhesion, recruitment of monocytes, and polarization of macrophages were investigated. NK cells-suppression effects of TAMs and CAFs were evaluated, in vivo experiments were used to further confirm the overall effects. It was found that CAFs attracted monocytes by secretion of IL-8 and promoted adhesion between monocytes and CRC cells by secretion of IL-6. Moreover, CAFs promoted M2 polarization of macrophages, which synergized with CAFs suppression function rather than recruitment of NK cells. The results in this study confirmed that TAMs and CAFs were synergetic and had the capacity to regulate NK cells in CRC and presented a novel mechanism for the effect.

## Results

### VCAM-1 overexpression is associated with TAMs enrichment

**Fig. 1:**
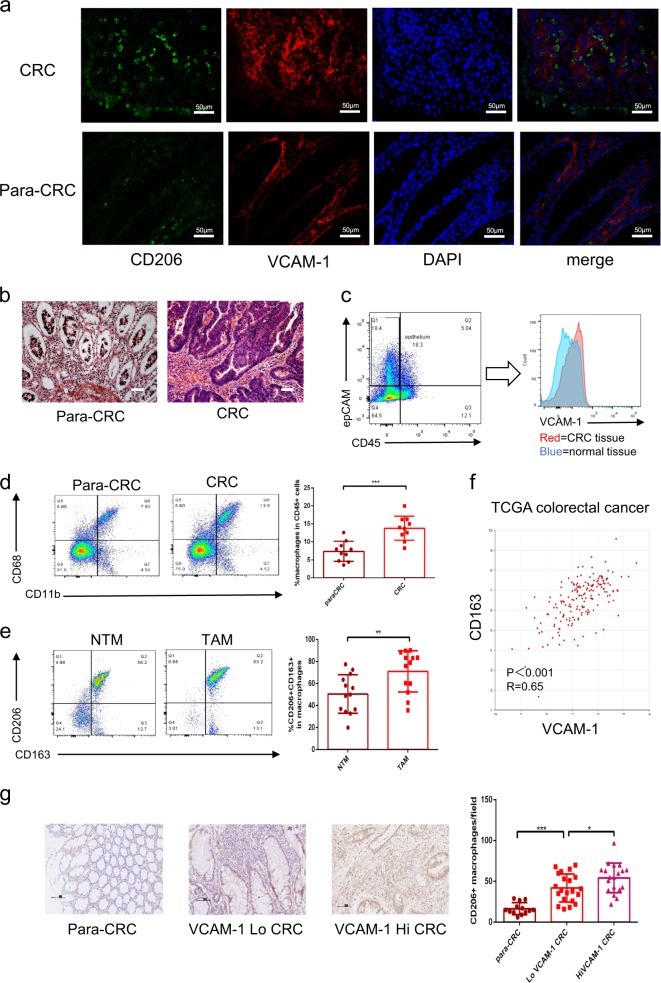
VCAM-1 overexpression is associated with TAMs enrichment. **a** Immunofluorescence of CD206 (green) and VCAM-1 (red) expression of CRC tissue and para-CRC (para-colorectal cancer) normal tissue (magnification, ×400, scale bar: 50 μm), nuclei were visualized by DAPI. **b** HE stain of para-CRC normal tissue and CRC tissue. (magnification, ×200, scale bar: 100 μm). **c** FACS analysis of VCAM-1 expression in the epithelium or CRC cells which gated as CD45- epCAM+. **d** FACS analysis and quantification of CD11b (+) CD68 (+) macrophages in human CRC tissue and para-CRC normal tissue. **e** FACS analysis and quantification of CD163 (+) CD206 (+) cells in normal tissue macrophages (NTMs) and TAMs. **f** VCAM-1 expression was positively associated with CD163 in clinical colorectal cancer specimens (*R* = 0.65, *P* < 0.001). Gene correlation analysis was based on the TCGA colorectal cancer dataset and was analyzed via the R2: Genomics Analysis and Visualization Platform. **g** IHC analysis and quantification of CD206 expression in para-CRC normal tissue, VCAM-1 low expression CRC tissue, and VCAM-1 high expression CRC tissue (scale bar: 100 μm). Error bars represent mean ± s.d.; **P < 0.01; ***P < 0.001; n.s. not significant; by unpaired two-sided Student’s *t*-test (**b**, **c**, **e**)

As shown in Fig. 1a, high VCAM-1 expression and CD206 (+) macrophages infiltration were observed in cancer tissues compared with normal tissue in CRC, these findings were further analyzed by flow cytometry. To exclude vascular endothelium and other hemocytes which usually express VCAM-1, the epithelial cells were gated as CD45− epCAM+ subpopulation. The results showed that the expression of VCAM-1 in CRC cells was higher compared with normal epithelium (Fig. 1c). This assay confirmed the enrichment of macrophage (CD11b+CD68+) in cancer tissue (Fig. 1d). Moreover, higher infiltration of CD163+CD206+ TAM was observed (Fig. 1e). To explore whether VCAM-1 expression was associated with TAM infiltration, TCGA colon adenocarcinoma was used to examine the correlation between CD163 and VCAM-1 via the R2: Genomics Analysis and Visualization Platform (http://r2.amc.nl). As shown in Fig. 1f, CD163 expression was positively associated with VCAM-1 expression in colon adenocarcinoma, and the IHC analysis and quantification in clinical specimen also showed the positive correlation between CD206 and VCAM-1 expression (Fig. 1g), which showed TAM infiltration in VCAM-1 overexpression CRC tissue was higher compared with VCAM-1 low expression CRC tissue or normal tissue. These results point to a possible association between VCAM-1 expression in cancer tissue and M2 macrophages infiltration.

### CC-CAFs promote adhesion between monocytes and CRC cells through VCAM-1/IL-6

**Fig. 2:**
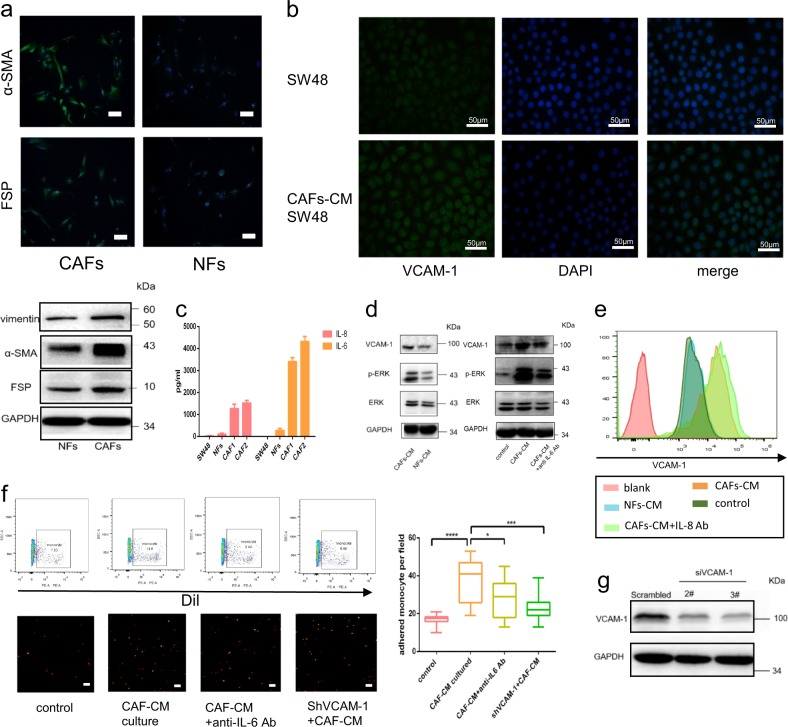
CC-CAFs promote adhesion between monocytes and CRC cells through VCAM-1/IL-6. **a** Immunofluorescence of α-SMA and FSP expression in CAFs and NFs (magnification, ×400, scale bar: 50 μm), nuclei were visualized by DAPI. Western-blot of α-SMA and vimentin and FSP in CAFs and NFs. **b** Immunofluorescence of VCAM-1 expression in SW48 cells with or without 24 h cultured in CAFs-CM (magnification, ×400, scale bar: 50 μm). **c** SW48 cells cultured in CAFs-CM or NFs-CM for 24 h, protein expression was determined by western blotting and representative results from one of the three independent experiments are presented. **d** Quantitative analysis of IL-6 and IL-8 levels by enzyme-linked immunosorbent assay (ELISA). The conditioned media from the cultured CC-CAFs derived from two different individuals (CAF1 and CAF2) and SW48 cells and para-cancer tissue-derived NFs (normal fibroblasts) were collected to detect the level of IL-6 and IL-8, representative results from one of the three independent experiments are presented. **e** FACS analysis of VCAM-1 expression in SW48 cells with or without treatment with CAFs-CM or NFs-CM. **f** Monocytes-Dil which adhered with SW48 or SW48-shVCAM-1 was observed by fluorescence microscopy (bottom, magnification, ×100, scale bar: 50 μm), the monocytes were quantified by flow cytometry (top), *n* = 6. Error bars represent mean ± s.d.; **P < 0.01; ***P < 0.001; n.s. not significant; by unpaired two-sided Student’s *t*-test. **g** Effect of VCAM-1 knockdown in SW48 was evaluated by western-blot

As shown in Fig. 2a, CAFs and normal fibroblasts (NFs) were isolated from human CRC tissues and para-cancer normal tissues. Western blot and immunofluorescence were used to identify the phenotype of CAFs as higher expression of α-SMA, FSP, and vimentin compared with NFs. Effects of CAFs on CRC cells line SW48 were investigated using CAFs-CM culture system. Figure 2b shows that VCAM-1 was up-regulated after cultured in CAFs-CM based on immunofluorescence, which was further confirmed by flow cytometry (Fig. 2e). To identify the cytokine which promoted enhanced VCAM-1 expression, CAFs and NFs were cultured in serum-starved F12 for 24 h, the level of IL-6 and IL-8 were determined by ELISA. As shown in Fig. 2d, the level of IL6 and IL8 was significantly higher in CAFs-derived condition medium (CM) compared with that presented in NFs-derived CM, but SDF-1 was not detected in the CM derived from CC-CAFs (data not shown). We further investigated the cytokine involved in the effects of CAFs-CM. As shown in Fig. 2c, e, CAFs-CM rather than NFs-CM increased the phosphorylation of ERK1/2 and up-regulated VCAM-1 expression. However, the CAFs-induced effects were partly inhibited after the application of anti-IL-6 antibody, but not when anti-IL-8 antibody was administered. These results suggested that IL-6 rather than IL-8 expressed in CAFs-CM up-regulated VCAM-1 expression in CRC cells. Based on clinical evidence, this study sought to establish whether the CAFs-induced VCAM-1 up-regulation is associated with macrophages infiltration. To test this possibility, monocyte-CRC cell adhesion assay was conducted. As shown in Fig. 2f, CRC cells cultured in CAFs-CM significantly enhanced the adhesion between CRC cells and monocytes, but this CAFs-mediated adhesion was impaired when anti-IL6 antibody was administered or after transfection with 3# siRNA of VCAM-1, the effect of siRNA was confirmed by western-blot (Fig. 2g). In summary, these results indicated that CAFs promoted monocytes adhesion by up-regulating VCAM-1 expression and IL-6 secretion.

### CC-CAFs attract monocytes via IL-8/CXCR2 pathway thereby inducing M2 polarization of macrophages

**Fig. 3:**
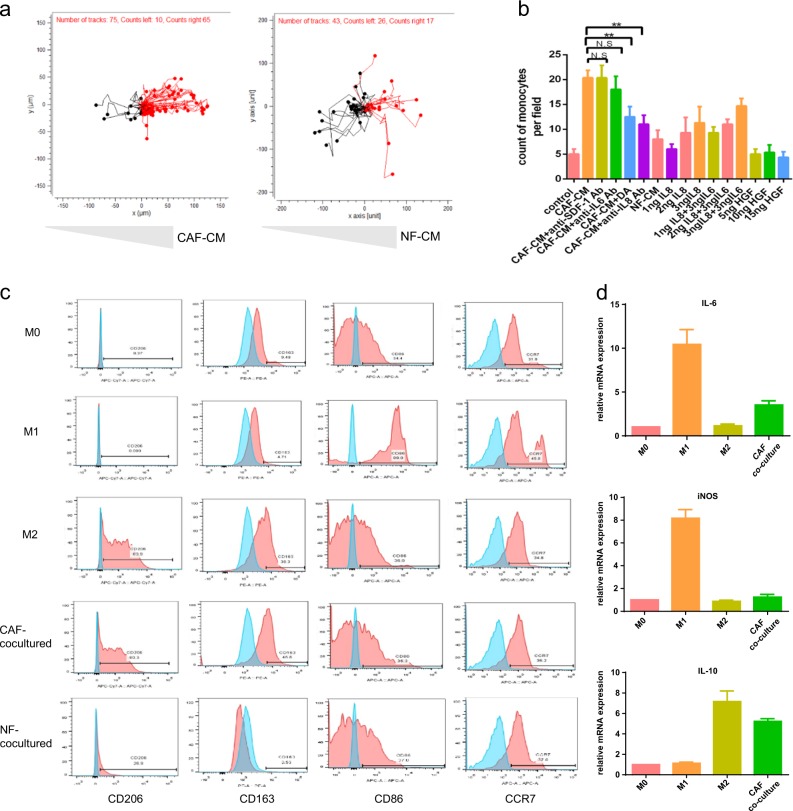
CC-CAFs attract monocytes via IL-8/CXCR2 pathway and subsequently induce M2 polarization of macrophages. **a** Migration plots of monocytes in CAFs-CM or NFs-CM gradient. The trajectory of monocytes migration was shown, the cells migrated towards condition medium was marked in red. **b** Quantification of migrated monocytes. (*n* = 3), DA (danirixin). **c** CD206, CD163, CD86, and CCR7 expression in macrophages was analyzed by flow cytometry (red), cell without stain serves as a negative control (blue). **d** mRNA expression of IL-6, iNOS, and IL-10 in macrophages. (*n* = 5), GAPDH was used as a control. Error bars represent mean ± s.d.; **P < 0.01; ***P < 0.001; n.s. not significant; by unpaired two-sided Student’s *t*-test (**b**, **d**)

Given that TAMs infiltration was enhanced in cancer tissue, we tested whether CAFs can influence the chemotaxis of monocytes. To determine the effect of CAFs on monocytes recruitment, cell chemotaxis assay was performed in μ-Slide Chemotaxis ibiTreat. As shown in Fig. 3a, monocytes were more attracted to CAFs CM than to the control medium, but this effect was not observed in NFs-CM. The underlying mechanism was then investigated by 24-well Transwell chambers. After the application of danirixin, a CXCR2 antagonist, the CAFs-CM-induced chemotaxis of monocytes was impaired. Similar results were observed after the administration of the anti-IL-8 antibody. Moreover, the administration of recombinant IL-8 in the lower chamber promoted chemotaxis of monocytes in a dose-dependent manner, which was not observed when IL-6 or HGF was applied. Only minimal enhancement of chemotaxis was observed after the co-administration of IL-6 and IL-8 compared with IL-8 alone (Fig. 3b). These results indicated that IL-8 rather than IL-6 present in CAFs-CM attracted monocytes. The effects of CAFs on macrophage polarization were then examined, after a 72 h co-culture of CAFs with macrophages, the expression of CD206, CD163 in the macrophages increased whereas that of CD86 and CCR7 was not altered (Fig. 3c), these effects were CAFs-specific, as it was not observed in NFs co-culture group. Moreover, mRNA expression of IL-10, a marker of M2 macrophages, increased after co-culturing with CAFs, but no detectable changes were observed in mRNA expression of M1, a marker of IL-6 and iNOS (Fig. 3d). These datasets indicated that co-culturing with CAFs promotes typical M2 polarization of the macrophages. These findings suggested that human CC-CAFs attracted monocytes through IL-8/CXCR2 pathway and subsequently contributed to M2 polarizations.

### CC-CAFs-induced macrophages promote migration of CRC cells and suppress NK cells killing ability

**Fig. 4:**
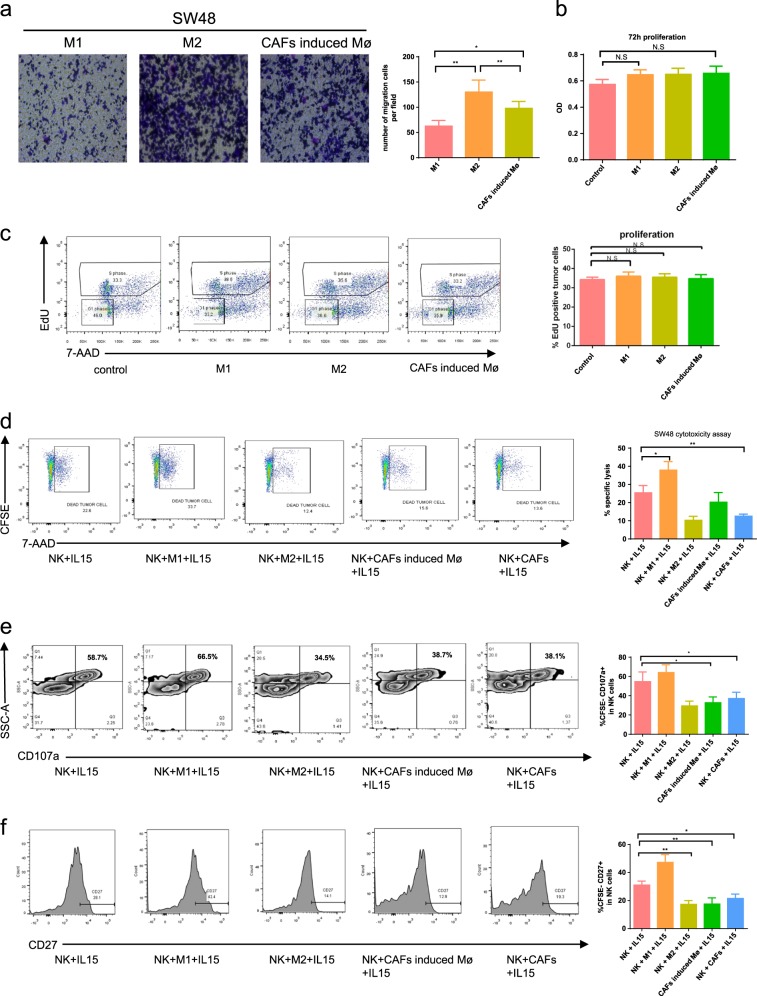
CC-CAFs-induced macrophages promote migration of CRC cells and suppress NK cells function. **a** Transwell migration assay of SW48 cells induced by macrophages (magnification, ×100). The blue dot in picture indicated migrated tumor cells. **b** Proliferation of SW48 cells induced by macrophages was evaluated by CCK-8 assay. (*n* = 3). **c** Proliferation of SW48 cells induced by macrophages was evaluated by EdU incorporation experiment (*n* = 3), the proportion of EdU positive cells was quantified. CAF induced Mφ refers to macrophages induced by CAFs. **d** FACS analysis and quantification of SW48 cells killed by NK cells (*n* = 5). **e** FACS analysis of CD107a (+) cells in NK cells. **f** FACS analysis of CD27 (+) cells in NK cells. Error bars represent mean ± s.d.; **P < 0.01; ***P < 0.001; n.s. not significant; by unpaired two-sided Student’s *t*-test (**a**–**f**)

The effects of CAFs-induced macrophages on CRC cells were evaluated. As shown in Fig. 4a–c, CAFs-induced macrophages promoted the migration of CRC cells whereas there they did not have any significant effect on proliferation. The effects of CAFs-induced macrophages on NK cells were then assessed to determine the effect of CAFs-induced macrophages on NK cells. NK cytotoxicity and activation after incubation with CAFs-induced macrophages were evaluated. The cytotoxicity assay was performed on NK cells, which were pre-co-cultured with CAFs or polarized macrophages, with CFSE targeted SW48 cells serving as target cell line. Dead tumor cells were analyzed by flow cytometry as CFSE+ 7AAD+. Co-culture of NK cells with M1 macrophages increased the proportion of 7AAD+ SW48 cells to approximately 30%. However, co-culture with M2 macrophages or CAFs-induced macrophages reduced the cytotoxicity of NK cells to 12% and 15%, respectively. Interestingly, decreased cytotoxicity of NK cells which were incubated with CAFs was observed (Fig. 4d). To further confirm if the changes in cytotoxicity were due to alternation in the activation level of NK cells, expression of CD27, a marker of NK cells activation, and that of CD107a, a marker of degranulation, were evaluated. In Fig. 4d, NK cells were analyzed by flow cytometry as CFSE-CD107a+. The addition of M2 macrophages, CAFs-induced macrophages or CAFs reduced levels of CD107a, whereas CD107a expression in M1 macrophages was increased compared with IL-15 activated NK cells. Similar results were observed in CD27 expression (Fig. 4e). These results demonstrated that CAFs-induced macrophages promoted the migration of CRC cells and protected CRC cells from NK cells-mediated killing by suppressing NK cells function.

### CAFs promote CRC progression partly by enhancing TAMs infiltration in vivo

**Fig. 5:**
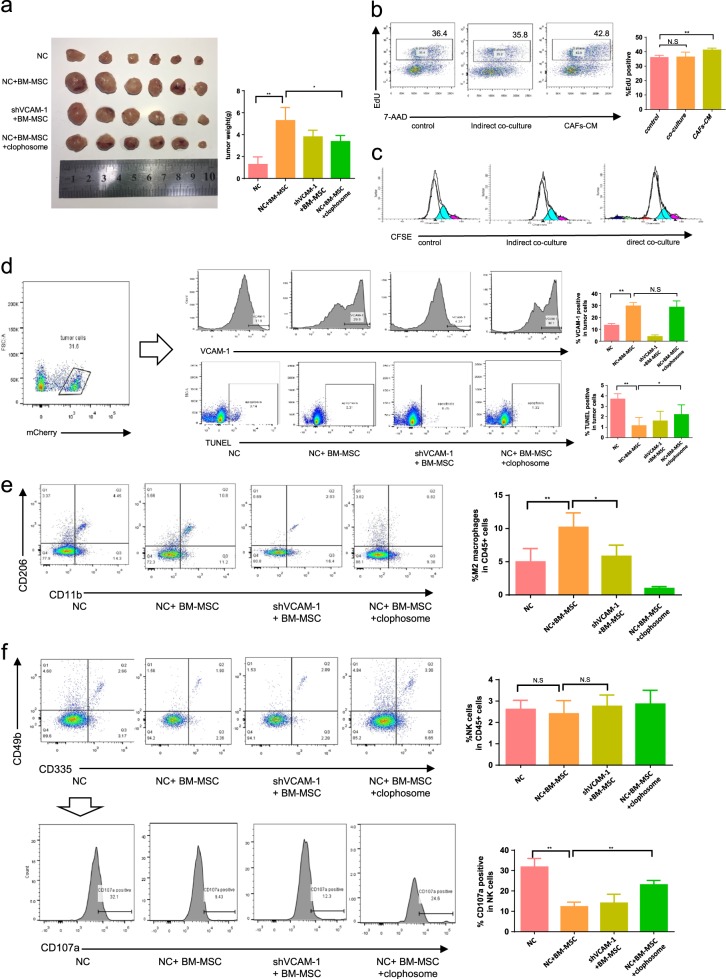
CAFs promote progression of CRC cells partly through the promotion of TAMs infiltration in vivo. **a** Subcutaneous tumor from CT-26 cells or CT-26-shVCAM-1 injected into the flank of Balb/c mice with or without BM-MSC and clophosome. The weight of subcutaneous tumor was quantified (*n* = 6). **b** Proliferation of SW48 cultured with or without CAFs-CM was evaluated by EdU incorporation experiment, the proportion of EdU positive cell was quantified (*n* = 3). **c** Proliferation of SW48 indirect or direct co-cultured with CAFs was evaluated by CFSE. **d** FACS analysis of VCAM-1 expression (upper) and apoptosis rate (bottom) in mCherry-targeted tumor cells (*n* = 6). **e** FACS analysis of CD206(+) CD11b (+) macrophages in subcutaneous tumor (*n* = 6). **f** FACS analysis of NK cells in subcutaneous tumor and expression of CD107a in NK cells (*n* = 6). Error bars represent mean ± s.d.; **P < 0.01; ***P < 0.001; n.s. not significant; by unpaired two-sided Student’s *t*-test (**a**, **b**, **d**–**f**)

Xenograft model was used to determine the role of CAFs in CRC in vivo. CT26-mCherry alone, CT26- mCherry mixed with BM-MSC, and shVCAM-1-CT26- mCherry mixed with BM-MSC were injected into the flanks of Balb/c mice. After the 1st week, 6 randomly selected mice from CT26-mCherry mixed with BM-MSC group were injected via I.V. route with clophosome to deplete the macrophages. The mice were then sacrificed after 3 weeks. As shown in Fig. 5a, b, the tumors produced by co-injection of CT26-mCherry mixed with BM-MSC were significantly larger than those produced by CT26-mCherry alone. In contrast, VCAM-1 knocking down or administration of clophosome abolished this effect. To explain the pro-tumor effect of CAFs after the depletion of macrophages or VCAM-1 knockdown, the effect of CAFs on the proliferation of CRC cells was investigated using the EdU incorporation assay. The assay revealed that CAFs-CM treatment enhanced the proliferation of CRC cells, but not in transwell co-culture (Fig. 5b). These results suggested that the inhibition effect of CAFs on cell proliferation was increased compared with CM culture due to additional crosstalk between two cells type in transwell system. We further confirmed this effect, SW48 cells were directly and indirectly co-cultured with CAFs, the proliferation rate was evaluated by CFSE staining, and the tumor cells were gated as CFSE+. Enhanced proliferation of SW48 cells was observed after being directly co-cultured with CAFs but this effect was not observed after indirect co-culturing with CAFs within 48 h (Fig. 5c). These results indicated that CAFs can promote the proliferation of CRC cells in direct contact manner, and it can also indirectly promote proliferation in certain conditions but may not predominant in the presence of crosstalk between cancer cells and CAFs.

Single-cell suspension of subC tumor was analyzed by flow cytometry to investigate the mechanisms responsible for the changes in tumor volume. The tumor cells were gated as mCherry+, then the expression of VCAM-1 and apoptosis rate were analyzed. As shown in Fig. 5d, VCAM-1 expression in CT26-mCherry mixed with BM-MSC group was up-regulated compared with CT26-mCherry alone. As postulated in this study, the proportion of apoptosis in the tumor cells, which were gated as TUNEL+ mCherry+, was decreased after co-injection of BM-MSC, but these effects of BM-MSC were eliminated after the depletion of macrophages or VCAM-1 knockdown. M2 macrophages were gated as CD45+ CD11b+ CD206+. Figure 5e shows that clophosome depleted M2 macrophages increased M2 macrophages infiltration in CT26-mCherry mixed with BM-MSC group compared with CT26-mCherry alone, and low infiltration was observed after VCAM-1 knocked down in tumor cells. An attempt was made to quantify the infiltration of NK cells which were gated as CD45+ CD335+ CD49b+. Figure 5f shows that no significant difference was observed among the experimental groups. However, the expression of CD107a in the infiltrated NK cells was decreased following BM-MSC co-injection, and the cytotoxicity of NK cells was partly abolished after the knockdown of VCAM-1 or macrophages depletion. In summary, CAFs promoted CRC progression partly by promoting TAMs infiltration and up-regulation of VCAM expression in vivo. CAFs also promoted the proliferation of CRC cells mainly through direct contact.

## Discussion

Solid tumor grows in complicated microenvironments including in the presence of CAFs, macrophages, neutrophils, and extracellular matrix^20^. Recent studies have shown that CAFs actively secret cytokines such as HGF, IL6, and SDF-1 which have positive effects on the cancer^1,5,7,8,21,22^ and the critical roles of TAMs in tumor progression are well documented^23,24^. CAFs and TAMs suppress antitumor immune responses^13,25^, and function synergistically during tumor progression^26^. However, their relationship during tumor progression has not been elucidated. Here, we report a novel role of human CC-CAFs relating to its ability to promote M2 macrophages polarization and recruitment by up-regulating VCAM-1 expression in CRC cells and secreting chemotactic factor and inhibiting NK cells function. Firstly, it was found that M2 macrophages infiltration was increased in cancer tissues, and the VCAM-1 expression and CD206+ macrophages infiltration were correlated based on immunohistochemical analysis. Secondly, culturing of CRC cells with CAFs showed increased adhesion property with monocytes, and this effect was partly abolished by the administration of anti-IL6 antibody or knockdown of VCAM-1. Thirdly, using chemotaxis assay, the CAFs-derived CM attracted monocytes and subsequently promoted M2 polarization, the chemotaxis effect was reduced by CXCR2 antagonist. Importantly, in vivo experiment showed that CAFs promoted M2 macrophages recruitment. Finally, M2 macrophages and CAFs-induced macrophages suppressed NK cells function, and depletion of macrophages in cancer tissue partly compromised the CAFs-induced tumor progression.

It has recently been reported that VCAM-1 expressed on the surface of the tumor is capable of interacting with VLA4 on monocytes^27,28^, and overexpression of VCAM-1 is associated with macrophages infiltration^28,29^, which creates a suitable tumor microenvironment, enhances invasion, and suppresses the immune response. Chen et al.^30^ reported that VCAM-1 mediated the binding of TAMs to cancer cells in breast cancer, which was similar to the results of this study, in which CAFs-derived IL-6 increased adhesion between CRC cells and monocytes by enhancing VCAM-1 expression. Moreover, animal experiments where mCherry-targeted tumor cells were quantified by flow cytometry to exclude endothelial cells which actively express VCAM-1, confirmed that CAFs can promote VCAM-1 expression in cancer cells and macrophages recruitment. CAFs-induced macrophages infiltration was suppressed when VCAM-1 was knocked down in tumor cells, indicating an important role of VCAM-1 in CAFs-induced TAMs recruitment.

Chemokines are associated with the recruitment of leukocytes in tumor^31–34^. Cytokines like CCL2, and SDF-1 are expressed in tumor stroma, and strong evidence indicates that these cytokines are associated with TAM accumulation^35^. This study demonstrated that human CC-CAFs significantly attracted monocytes. Previously, Comito, G et al.^26^ reported that CAFs-derived SDF-1 contributes to monocyte recruitment in prostate fibroblasts, however, in the present study, instead of SDF-1, which was not detected in the supernatant of human CC-CAFs, CXCL8 or IL-8 were abundant in the secretion of CAFs, presumably due to the difference in source of CAFs. IL-8 can attract monocytes in a dose-dependent manner, and CXCR2 antagonist can partly block CAFs-induced chemotaxis of monocytes, which indicates that CAFs-derived IL-8, rather than SDF-1, contributed to monocytes attraction in human CRC. TAM can be subdivided into classically activated (M1) and alternatively activated (M2) macrophages. M2 was reported to promote tumor progression and suppression of immune response^36^. It was observed that co-culture with CAFs promoted M2 polarization of monocytes, and CAFs-induced TAMs suppressed NK cells function by reducing NK cells activation. In this research, a novel tumor-oriented mechanism which suppressed NK function by cross-talk between CAFs and M2 polarized macrophages was proposed. However, further investigations are required to validate this mechanism.

**Fig. 6:**
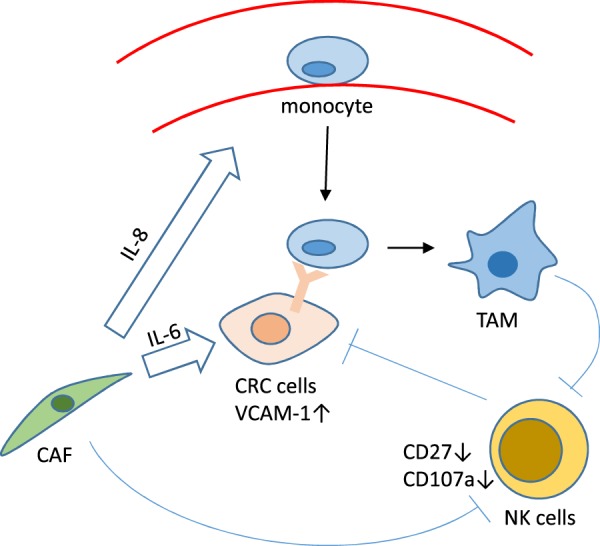
Proposed mechanism of CAFs mediates TAMs infiltration and subsequently suppresses NK function. Based on our results, we proposed a model involving immune suppression in CRC. CC-CAFs produce IL-8, which recruits monocytes and produce IL-6 to promote the expression of VCAM-1 in CRC cells and enhance monocytes adhesion. CC-CAFs promoted polarization of M2 macrophages, which synergized with CAFs suppress the function of NK cells

NK cells are among the major cell types of the innate immune system, which kill their target cells without MHC restriction or prior sensitization^16^. NK cells dysfunction promotes tumor progression in several types of human solid tumor^37–39^. Hayakawa et al.^40^ illustrated that CD27 low NK cells have a higher threshold of being active compared with CD27 high NK cells. Krneta et al.^13^ demonstrated that M2 macrophages reduce CD27 and impair NK cells killing of lymphoma cells. Similarly, we found that M2 and CAFs and CAFs-induced TAMs impaired NK cells killing of CRC cells. Moreover, in vivo experiments showed that CAFs-induced tumor progression was compromised when macrophages were depleted by the administration of clophosome. However, the NK cells infiltration was not changed in this study, which indicated that CAFs can induce M2 macrophages infiltration and subsequently impair NK cells activation, rather than recruitment (Fig. 6).

In brief, the results in this work indicate that human CC-CAFs promotes TAMs infiltration and M2 macrophages polarization, which subsequently impairs the NK cells function.

## Materials and methods

### Ethic statement

CRC tissues were obtained from patients who had undergone surgery at the Tongji Hospital, Huazhong University of Science and Technology. Written informed consents were obtained from all participants, and all procedures were authorized by the Ethical Committee of Tongji Hospital.

### Isolation of CAFs and TAMs from cancer tissues and subsequent primary culture and CM collection

Fresh colorectal adenocarcinoma tissues and para-cancer normal tissue were obtained from patients who have undergone surgery, tumor tissue specimens were pathologically confirmed as colorectal adenocarcinoma, while the “para-CRC” was confirmed as having no cancerous tissue invasion by two pathologists from the Tongji Hospital, Huazhong University of Science and Technology. CC-CAFs and primary colorectal NFs were isolated from CRC tissues and adjacent normal tissue as our previous work. Briefly, the specimen was cut into sections, then subjected to collagenase IV (Invitrogen, CA, USA) digestion for 3 h at 37 °C. After digestion, it was passed through a 70 µm mesh (BD Falcon, CA, USA). We centrifuged the filtrate and cultivated the cells in red blood cell lysis buffer to eliminate red blood cells, washed the remaining cells with PBS. The cells for analysis were re-suspended in FACS staining buffer for flow cytometry, cells for culturing were then plated in Dulbecco’s modified Eagle’s medium (DMEM) with low glucose content containing 10% fetal bovine serum (FBS; Gibco, USA) and 1% penicillin/streptomycin, adherent cells were removed to other culture dish for cultivation of CAFs and NFs. For the collection of CM, 2 × 10^6^ cells were seeded onto 10-cm plate and cultured for 36 h, the medium was replaced with 5 ml fresh F12 without serum and cells were cultured for further 24 h, the CM was collected and filtered, stored at −40 °C.

### Flow cytometry stain and analysis

The cells were stained with following antibodies (BD Bioscience, Heidelberg, Germany) according to the instruction of manufacturers: anti-CD45, anti-CD206, anti-CD163, anti-CD11b, anti-CD335, anti-CD49b, anti-CD197, anti-CD64, anti-CD68, anti-CD56, anti-CD107a, anti-CD27, anti-CD106. Labeled cells were analyzed on BD FACSVerse Flow Cytometer, and the data were processed using FLOWJO software (Treestar).

### Transwell co-culture system

For co-culture of CRC cells and CAFs, CAFs were seeded in the upper insert of a six-well Transwell apparatus (0.4 μM pore size, Corning, Lowell, MA) while CRC cell line in the lower chamber. After 48 h, both CAFs and CRC cells were then harvested for further biochemical analyses. In the co-culture experiments of CAFs and macrophages, macrophages induced by 320 nM PMA were seeded in lower chamber and CAFs or NFs seeded in upper chamber. After 72 h of co-culture, both CAFs and differentiated macrophages were harvested for further analyses.

### M1 and M2 macrophage polarization

Human polarized macrophages were prepared from peripheral blood monocyte cells (PBMC) of healthy donors. Briefly, human peripheral blood mononuclear cells were isolated by using density gradient centrifugation. Monocytes were isolated using anti-CD14 microbeads (Miltenyi Biotech, Germany). Macrophages were obtained by 6 days culture of monocytes in RPMI 1640 containing 10% FBS and 1% penicillin/streptomycin and 100 ng/ml macrophage colony-stimulating factor. For macrophages polarization, cells were cultured with the addition of 100 ng/ml LPS and 20 ng/ml IFN-γ (for M1 polarization) or 20 ng/ml IL-4 plus 20 ng/ml IL-13 (for M2 polarization) for 48 h. In some experiments, macrophages were co-cultured with CAFs using Transwell chambers (Corning, MA, USA) for 3 days.

### Cell lines, reagents, transfections

We obtained cell lines (CT26, SW48, and NK-92) from the American Type Culture Collection. The human CRC cell line SW48 was incubated in DMEM with high glucose content (Invitrogen, CA, USA) containing 10% FBS and 1% penicillin/streptomycin, murine CRC cell line CT26 was incubated in RPMI 1640 (Invitrogen, CA, USA) containing 10% FBS and 1% penicillin/streptomycin. The cells were cultured at 37 °C with 5% CO_2_ and 95% air. Danirixin (MedChem Express, USA), a specific chemical inhibitor of CXCR2; Anti-IL-6 antibody (Sino Biological, China), a neutralizing antibody for IL-6, recombinant human IL-8, IL-6, HGF (PeproTech, USA). VCAM-1 shRNA expression and shRNA control lentiviral particle were purchased from GeneChem Co. (Shanghai, China). For lentiviral transduction, 5000 cells/well were seeded in 96-well tissue culture plates and infected the following day with lentiviral particles at a MOI of 10 in the presence of 10 mg/ml polybrene, purchased from Santa-Cruz Biotechnology (Dallas, TX). After infection, CT26 was selected with 7 μg/ml puromycin, purchased from Life Technologies (Carlsbad, CA).

### Immunofluorescence assay

The specific biomarkers of CAFs, α-SMA, FSP, and vimentin expression induced by the CAFs in SW48 cells were determined by immunofluorescence assay. Cultured SW48 cells in the chambers were washed with PBS, fixed with 4% paraformaldehyde for 10 min, and then permeabilized in 0.1% Triton X-100 for 25 min. After being blocked with goat sera at 37 °C for 30 min, the cells in the chambers were treated with anti-α-SMA, anti-FSP, or anti-VCAM-1 (Proteintech, China) at 4 °C overnight. After being washed, the bound antibodies were detected with suitable DyLight 594 red-conjugated or FITC green-conjugated secondary antibodies and photo imaged under a fluorescent microscope.

### Patient and specimen preparation

The CRC primary tumor and para-tumor normal tissue were acquired from 50 patients who have undergone surgery. Then the expression of VCAM-1 and CD206(+) macrophages were observed by immunohistochemistry after frozen section.

### Static adhesion assay

Primary human monocytes were labeled with 5 μM Dil (Beyotime Biotech, China) following the instructions of the manufacturer. 5 × 10^5^ SW48 cells with or without cultured in CAFs-CM were plated onto 6-well plates. The medium was removed from the wells, and 5 × 10^5^ Dil-labeled monocytes were added to monolayer SW48 cells. After incubated for 30 min at 37 °C, the unattached cells were gently washed twice with 10% FBS-containing DMEM, the count of monocytes adhered to SW48 was examined under fluorescence microscopy. Then cells were digested with 0.25% trypsin, the adhered monocytes were quantified by flow cytometry (BD, FACSVerse). The ratio of adhered monocytes to tumor cells was calculated as the ratio of monocytes to 5 × 10^5^ cells.

### Quantitation of IL-8, IL-6, and SDF-1 by enzyme-linked immunosorbent assay (ELISA)

Conditioned medium from the CC-CAFs was collected and IL-8, IL-6, and SDF-1 ELISA kit (eBioscience, CA, USA) were used to determine the level of IL-8, IL-6, and SDF-1 in CC-CAFs. All tests were performed in duplicate. The plate was read at a wavelength of 450 nm. The concentration of cytokine (pg/ml) was defined by the standard curve. F12 without supplements served as the control.

### Western blotting

Western blotting was carried out according to the standard protocols described previously. We used primary antibodies raised against GAPDH (Santa-Cruz Biotechnology, CA, USA), p-ERK (Cell Signaling Technology, MA, USA), α-SMA, FSP (Proteintech, China). Goat anti-mouse and anti-rabbit antibodies conjugated with horseradish peroxidase were used as secondary antibodies (Jackson ImmunoResearch, PA, USA), and we detected the blots using enhanced chemiluminescence (ECL) (Dura, Pierce, NJ, USA).

### RNA extraction and real-time polymerase chain reaction (PCR) assay

Total RNA was extracted using TRIzol Reagent (Invitrogen, CA, USA) following the manufacturer’s protocol and was reverse-transcribed into complementary DNA (cDNA) using a Superscript Reverse Transcriptase Kit (Transgene, France). Super SYBR Green Kit (Transgen, France) was used to carry out real-time PCR in ABI7300 real-time PCR system (Applied Biosystems).

### Migration assay

We investigated the migration of SW48 cells using Transwell chambers (Corning, MA, USA) in a 24-well plate containing 8-µm pores. Tumor cells in Serum-free F12/DMEM were placed in the upper chamber and M0, M1, M2, were placed in the lower chamber. After culturing for 24 h, the cells that had migrated towards to lower chamber were fixed with 4% paraformaldehyde for 10 min, stained with crystal violet, and counted by bright-field microscopy. Chemotaxis assay was performed in 24-well plate containing 5-µm pores, 2 × 10^5^ monocytes were loaded into the upper chamber, CAF CM, IL-6, IL-8, HGF, and danirixin were added to the lower chamber, after the cells were incubated for 24 h in 37 °C with 5% CO_2_ and 95% air, the number of cells in lower chamber was determined.

### 3D migration assay

Monocytes were seeded into μ-Slide chemotaxis (Ibidi, Munich, Germany) according to the instructions of the manufacturer. Migration directed towards CAFs-derived CM or NFs-derived CM was monitored with time-lapse Leica Microscope DMI6000 for 6 h. Cell tracking was performed using ImageJ Software. For the estimation of velocity and distance of the tracked cells, Chemotaxis and Migration Tool Software (Ibidi) was used.

### Detection of NK cells killing activity

For NK cells killing activity, 1 × 10^5^ polarized macrophages or CAFs were co-cultured with 1 × 10^5^ NK-92 cells for 24 h at 37 °C, then the NK-92 cells were added into 1 × 10^5^ SW48 cells which labeled with CFSE and incubated at 37 °C for 5 h. After the incubation, all cells were stained with 7-AAD (BD Bioscience), and CD107a (BD Bioscience). Cells were subsequently analyzed by flow cytometry.

### Animal experiment

Animal assays were performed according to Wuhan Medical Experimental Animal Care Guidelines. 6–7 weeks old BALB/c mice were bred under specific pathogen-free (SPF) conditions. The mice were divided into four randomized groups (*n* = 6 per group), 1 × 10^5^ CT-26-mCherry or CT-26-shVCAM-1 with or without BALB/c derived BM-MSC were subcutaneously injected into the flank of each mouse. After 7 days, one of the groups which injected with CT26-mCherry and BM-MSC were I.V. injected Liposomal clodronate (Encapsula NanoSciences, Brentwood, TN) (200 μL, twice a week) to deplete macrophages. The tumor size was measured using digital Vernier calipers every 3 days, the tumor volume was calculated by the following formula: volume = 1/2 × (width^2^ × length). All mice were sacrificed after 18 days, and the tumor was collected and visually examined. Then the tumor was cut into sections and subjected to collagenase IV (Invitrogen, CA, USA) digestion for 3 h at 37 °C. After digestion, it was passed through a 70 µm mesh (Miltenyi Biotech, Germany) and analyzed by flow cytometry.

### Public database and bioinformatics analysis

The gene expression correlations were revealed by the R2: Genomics Analysis and Visualization Platform (http://r2.amc.nl) using the TCGA colon adenocarcinoma dataset.

### Statistical analysis

Statistical analysis was performed with the use of GraphPadPrism software (San Diego, CA, USA). Two conditions were compared using an unpaired Student’s *t* test, 3 conditions were compared using 1-way ANOVA.

## References

[1] Damm S (2010). HGF-promoted motility in primary human melanocytes depends on CD44v6 regulated via NF-kappa B, Egr-1, and C/EBP-beta. J. Invest. Dermatol..

[2] Yan Y, Wang LF, Wang RF (2015). Role of cancer-associated fibroblasts in invasion and metastasis of gastric cancer. World J. Gastroenterol..

[3] Shiga K (2015). Cancer-associated fibroblasts: their characteristics and their roles in tumor growth. Cancers.

[4] Karakasheva TA (2018). IL-6 mediates cross-talk between tumor cells and activated fibroblasts in the tumor microenvironment. Cancer Res..

[5] Zhang X (2018). Human colorectal cancer-derived mesenchymal stem cells promote colorectal cancer progression through IL-6/JAK2/STAT3 signaling. Cell Death Dis..

[6] Cheng Y (2018). Cancer-associated fibroblasts induce PDL1+ neutrophils through the IL6-STAT3 pathway that foster immune suppression in hepatocellular carcinoma. Cell Death Dis..

[7] Ying L (2015). Cancer associated fibroblast-derived hepatocyte growth factor inhibits the paclitaxel-induced apoptosis of lung cancer A549 cells by up-regulating the PI3K/Akt and GRP78 signaling on a microfluidic platform. PloS One.

[8] Deying, W. et al. CAF-derived HGF promotes cell proliferation and drug resistance by up-regulating the c-Met/PI3K/Akt and GRP78 signalling in ovarian cancer cells. *Biosci. Rep.* **37**, BSR20160470 (2017). 10.1042/BSR20160470 PMC5469328 28258248

[9] Solinas G, Germano G, Mantovani A, Allavena P (2009). Tumor-associated macrophages (TAM) as major players of the cancer-related inflammation. J. Leukoc. Biol..

[10] Mantovani A, Sica A, Locati M (2007). New vistas on macrophage differentiation and activation. Eur. J. Immunol..

[11] Martinez FO, Helming L, Gordon S (2009). Alternative activation of macrophages: an immunologic functional perspective. Annu. Rev. Immunol..

[12] Zheng P (2018). Tumor-associated macrophages-derived exosomes promote the migration of gastric cancer cells by transfer of functional Apolipoprotein E. Cell Death Dis..

[13] Krneta T (2017). M2-polarized and tumor-associated macrophages alter NK cell phenotype and function in a contact-dependent manner. J. Leukoc. Biol..

[14] Zheng P (2017). Exosomal transfer of tumor-associated macrophage-derived miR-21 confers cisplatin resistance in gastric cancer cells. J. Exp. Clin. Cancer Res..

[15] Han Q, Shi H, Liu F (2016). CD163(+) M2-type tumor-associated macrophage support the suppression of tumor-infiltrating T cells in osteosarcoma. Int. Immunopharmacol..

[16] Vivier E (2011). Innate or adaptive immunity? The example of natural killer cells. Science.

[17] Zhang C (2017). Chimeric antigen receptor-engineered NK-92 cells: an off-the-shelf cellular therapeutic for targeted elimination of cancer cells and induction of protective antitumor immunity. Front. Immunol..

[18] Tang X (2018). First-in-man clinical trial of CAR NK-92 cells: safety test of CD33-CAR NK-92 cells in patients with relapsed and refractory acute myeloid leukemia. Am. J. Cancer Res..

[19] Parkhurst MR, Riley JP, Dudley ME, Rosenberg SA (2011). Adoptive transfer of autologous natural killer cells leads to high levels of circulating natural killer cells but does not mediate tumor regression. Clin. Cancer Res..

[20] Mao Y, Keller ET, Garfield DH, Shen K, Wang J (2013). Stromal cells in tumor microenvironment and breast cancer. Cancer Metastasis Rev..

[21] Kwon Y, Godwin AK (2017). Regulation of HGF and c-MET interaction in normal ovary and ovarian cancer. Reprod. Sci..

[22] Kalluri R (2016). The biology and function of fibroblasts in cancer. Nat. Rev. Cancer.

[23] Ostuni R, Kratochvill F, Murray PJ, Natoli G (2015). Macrophages and cancer: from mechanisms to therapeutic implications. Trends Immunol..

[24] Mantovani A, Sozzani S, Locati M, Allavena P, Sica A (2002). Macrophage polarization: tumor-associated macrophages as a paradigm for polarized M2 mononuclear phagocytes. Trends Immunol..

[25] Inoue T (2016). Cancer-associated fibroblast suppresses killing activity of natural killer cells through downregulation of poliovirus receptor (PVR/CD155), a ligand of activating NK receptor. Int. J. Oncol..

[26] Comito G (2014). Cancer-associated fibroblasts and M2-polarized macrophages synergize during prostate carcinoma progression. Oncogene.

[27] Liu YS (2017). MiR-181b modulates EGFR-dependent VCAM-1 expression and monocyte adhesion in glioblastoma. Oncogene.

[28] Qian BZ, Pollard JW (2010). Macrophage diversity enhances tumor progression and metastasis. Cell.

[29] Lu X (2011). VCAM-1 promotes osteolytic expansion of indolent bone micrometastasis of breast cancer by engaging α4β1-positive osteoclast progenitors. Cancer Cell.

[30] Chen Q, Zhang XH, Massague J (2011). Macrophage binding to receptor VCAM-1 transmits survival signals in breast cancer cells that invade the lungs. Cancer Cell.

[31] Germano G, Allavena P, Mantovani A (2008). Cytokines as a key component of cancer-related inflammation. Cytokine.

[32] Balkwill F (2004). Cancer and the chemokine network. Nat. Rev. Cancer.

[33] Mantovani A (2004). Chemokines in the recruitment and shaping of the leukocyte infiltrate of tumors. Semin. Cancer Biol..

[34] Bottazzi B (1983). Regulation of the macrophage content of neoplasms by chemoattractants. Science.

[35] Ueno T (2000). Significance of macrophage chemoattractant protein-1 in macrophage recruitment, angiogenesis, and survival in human breast cancer. Clin. Cancer Res..

[36] Van Overmeire E, Laoui D, Keirsse J, Van Ginderachter J, Sarukhan A (2014). Mechanisms driving macrophage diversity and specialization in distinct tumor microenvironments and parallelisms with other tissues. Front. Immunol..

[37] Sun C, Sun HY, Xiao WH, Zhang C, Tian ZG (2015). Natural killer cell dysfunction in hepatocellular carcinoma and NK cell-based immunotherapy. Acta Pharmacol. Sin..

[38] Takeda K (2001). Involvement of tumor necrosis factor-related apoptosis-inducing ligand in surveillance of tumor metastasis by liver natural killer cells. Nat. Med..

[39] Cerwenka A, Lanier LL (2001). Natural killer cells, viruses and cancer. Nat. Rev. Immunol..

[40] Hayakawa Y, Smyth MJ (2006). CD27 dissects mature NK cells into two subsets with distinct responsiveness and migratory capacity. J. Immunol..

